# Evaluation of pedicle screw accuracy and deviation from preoperative planning in intraoperative Cone-Beam Computed Tomography-Navigated lumbar spinal fusion: a prospective study

**DOI:** 10.1016/j.bas.2026.105988

**Published:** 2026-02-23

**Authors:** Gianluca Vadalà, Giuseppe Francesco Papalia, Niccolò Nardi, Fabrizio Russo, Luca Ambrosio, Girolamo Maltese, Rocco Papalia, Vincenzo Denaro

**Affiliations:** a Operative Research Unit of Orthopaedic and Trauma Surgery, Fondazione Policlinico Universitario Campus Bio-Medico, Via Alvaro del Portillo, 00128, Roma, Italy; b Research Unit of Orthopaedic and Trauma Surgery, Department of Medicine and Surgery, Università Campus Bio-Medico di Roma, Via Alvaro del Portillo, 00128, Roma, Italy

**Keywords:** Pedicle screw accuracy, Preoperative planning, Deviation, Spinal fusion, Navigation systems

## Abstract

**Introduction:**

Preoperative planning using navigation systems allows the surgeon to optimize pedicle screw placement, minimizing the risk of misplacement.

**Research question:**

This study aimed to assess the accuracy of pedicle screws placement, and the deviation of final position compared to preoperative planning using Cone-Beam Computed Tomography (CBCT) navigation system.

**Material and methods:**

A prospective observational study was conducted on patients who underwent lumbar spinal fusion with TLIF technique for degenerative spondylolisthesis using intraoperative imaging navigation system. Accuracy and superior facet joint violation (FJV) were assessed following Gertzbein and Robbins system (GRS) and Yson classification, respectively. Deviation from planning was calculated by linear, angular and 3D deviations between planned and implanted screws. The comparison of continuous variables by GRS was performed by ANOVA followed by Bonferroni's test. The association between vertebral level and screw deviations was evaluated using ANOVA analysis.

**Results:**

The study included 40 patients with a mean age of 62.8 years. Of 180 pedicle screws implanted, 177 (98.30%) were clinically acceptable (GRS A + B) and 97.50% were classified as Yson grade 0. ANOVA reported statistically significant results between GRS and tip deviations in lateral-medial (p = 0.005), antero-posterior (p = 0.01) and 3D (p = 0.003), and between GRS and head deviations in antero-posterior and 3D (p < 0.001). ANOVA demonstrated statistically significant results between vertebral level and accuracy (p < 0.001).

**Discussion and conclusion:**

The intraoperatively acquired image navigation system showed high accuracy. Screw deviation proved the guiding function of planning during screw insertion and demonstrated that for slight deviations no decrease in accuracy was found.

## Highlights

• Preoperative planning using navigation systems allows to optimize pedicle screw placement.
• The aim of this study was to assess accuracy of pedicle screws placement and deviation from preoperative planning.
• Intraoperative Cone-Beam Computed Tomography navigation system showed high accuracy of lumbar pedicle screws placement.
• Deviations between planned and implanted screws proved the guiding function of preoperative planning during screw insertion.

## 1 Introduction

Spinal fusion aims to allow the arthrodesis of one or more contiguous vertebrae (Wu et al., 2024). In recent years, in Europe and North America there has been a notable increase in the rate of use of spinal surgery, especially spinal fusion operations (Heck et al., 2023; Cortesi et al., 2017; Pannell et al., 2015). Life expectancy is an important factor related to this increasing phenomenon. Degenerative diseases have led to an increased demand for spinal fusion surgery (Sheikh et al., 2020). The increased demand and the need for extreme precision in spinal surgery have encouraged the development and using cutting-edge technological systems, such as image-guided intraoperative navigation (Vadalà et al., 2020; Mao et al., 2021). Intraoperative navigation systems are designed to maximize precision and accuracy in spine surgery and to reduce the mispositioning of the pedicular screws and the related complications (Vadalà et al.). These technologies are equipped with software that allows the preoperative planning of the implants (Papalia et al., 2024). Preoperative planning using navigation systems allows the surgeon to optimize pedicle screw placement by determining the ideal trajectory and screw dimensions, thus minimizing the risk of misplacement and related complications (Jiang et al., 2020; Mathew et al., 2013). Therefore, preoperative planning has been shown to significantly improve the accuracy of spinal instrumentation while reducing intraoperative screw revisions and radiation exposure (Van et al., 2015; Gubian et al., 2022). However, intraoperative considerations may require the surgeon to deviate from the preoperative pedicle screw placement planning. Moreover, measurement techniques for assessing pedicle screw placement have not yet clearly defined in thoraco-lumbar spine.

The first objective of this study is to evaluate the accuracy of pedicle screws implantation using a Cone-Beam Computed Tomography (CBCT) image-guided navigation system in a court of patients. The second objective is to evaluate the deviation of the final position of the pedicle screw compared to the pre-operative planning using a linear and angular measurement system. Finally, any correlations between these two parameters were sought to explain the role of pre-operative planning in spinal fusion surgery via an intraoperative navigation system.

## 2 Materials and methods

### 2.1 Patient population

A prospective observational study was conducted at the Orthopedics and Trauma Surgery Operative Research Unit of Fondazione Policlinico Universitario Campus Bio-Medico. The Ethical Committee of the Institution approved the study, which was performed in line with the principles of the Declaration of Helsinki. The inclusion criteria for this study were patients over 18 years of age, suffering from degenerative spondylolisthesis, undergoing lumbar spinal fusion surgery using intraoperative imaging navigation system since December 2021. Patients undergoing spinal fusion surgery for infections, trauma or tumours were excluded. Moreover, we excluded patients who did not have post-operative CBCT scan or patients having scans with metal artefacts. Spinal fusion surgery was performed in all patients using the TLIF technique, involving a midline skin incision for decompression and cage placement, along with percutaneous screw placement. Data extracted from the study population include age, sex, body mass index (BMI), vertebral levels and number of vertebral levels treated.

### 2.2 Navigation system and surgical setting

The navigation system used includes a mobile CBCT for the acquisition of surgical images (Loop-X, Brainlab), a central computer with navigation software (Curve Navigation, Brainlab) and a 4K display with a DICOM viewer, and a wide-field optical tracker that locates the reference frame positioned on the patient and the instrumentation equipped with reference matrices. The software enables the surgeon to perform pre-operative planning of pedicle screws and interbody cages. After positioning the reference frame and verifying detection with the optical tracker, intraoperative CT images are acquired. The images are loaded onto the computer and can be overlaid with preoperative MRI to achieve higher quality visualization Brainlab® Elements Spine Planning software is used to perform preoperative planning, allowing the surgeon to choose the size of the pedicle screw and interbody cage and manually position them on the CT scan.

### 2.3 Surgical procedure

Two paravertebral incisions of 1 – 2 cm are made on the sides of the vertebral segments to be treated. Once the vertebra is reached, the drill guide is used to locate the correct entry point of the screw into the vertebral pedicle and is positioned along the desired trajectory. Then, a neuro-monitoring clamp (NVM5, NuVasive) is attached to the drill guide, to allow real-time monitoring of electromyographic signals. During drilling, a k-wire is passed to confirm its placement in the pedicle cancellous bone. The screws are fixed using screwdrivers along the previously drilled holes. The intervention is completed with the positioning of rods of suitable size inserted inside specific guides placed on the heads of the screws. A final CBCT scan is performed to verify the implants' correct positioning.

### 2.4 Accuracy and superior facet joint violation

All patients in this study had a control CBCT scan performed at the end of surgery to evaluate the accuracy of each screw implanted. Accuracy was assessed following the “Gertzbein and Robbins system” (GRS) (Gertzbein and Robbins, 1990). For each screw grade B or lower, the orientation of the deviation in the mediolateral and cranio-caudal directions was recorded. The superior facet joint violation (FJV) was evaluated following the Yson classification (Yson et al., 2013). The FJV evaluation was performed for superior screws level (Liu et al., 2023).

### 2.5 Deviation from pre-operative planning

**Fig. 1:**
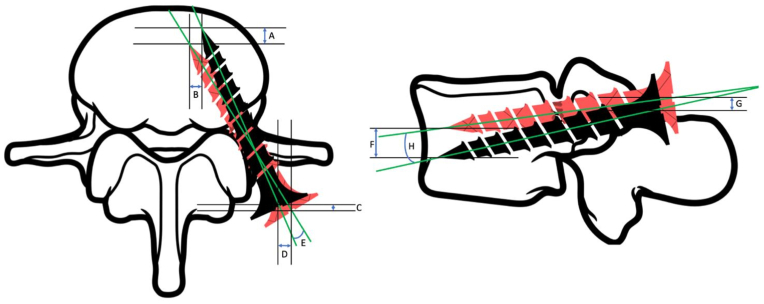
Calculation of deviation parameters from pre-operative planning.

The preoperative CBCT with planning and the postoperative control CBCT with the implant placed were used to calculate the deviation from preoperative planning. The calculation of the deviation parameters was carried out on the central computer of the navigation system. Measurements were performed by an experienced musculoskeletal radiologist blinded to the surgical planning and intraoperative results. Specialized software performed an overlap of the CBCT scans with a "curvature correction system" which guaranteed millimetric matching between the two scans. The parameters analyzed include the linear deviations of the tip and the entry point of the screw and the axial and angular deviations of the screw trajectory. The linear deviations were analyzed along the following three axes: on the axial projection in the anteroposterior direction and mediolateral direction, and on the sagittal projection in the craniocaudal direction. After having calculated the tip vector component and entry point on every single plane, following the formula "3D linear deviation = d(x)2 + d(y)2 + d(z)2″, the three-dimensional deviation was calculated (Jiang et al., 2020). The angular deviation was instead analyzed by calculating the angle formed by the central axis of the screw in its final position and by the central axis of the planned screw. The angular deviation was studied only two-dimensionally in the axial and sagittal planes (Fig. 1).

### 2.6 Analyzed variables

Statistical analysis was performed with Statistical Package for the Social Sciences (SPSS version 28.0). Continuous variables were reported with mean ± standard deviation (SD). The comparison of continuous variables with normal distribution of variance by GRS was performed by ANOVA followed by Bonferroni's test. The association between vertebral level and screw deviations was evaluated using ANOVA analysis. The statistical significance was established at p-value <0.05.

## 3 Results

### 3.1 Demographic and surgical data

A total of 40 patients suffering from degenerative spondylolisthesis and undergoing spinal fusion surgery with adequate control CT scans have been included in the following study. The population's average age was 62.6 ± 8.9 years; 18 patients were male (45%), and 22 patients were female (55%). The average BMI was 26.6 ± 3.5 (table). Spinal fusion was performed on a single level for 30 patients (75%) and on two levels for 10 (25%). A total of 180 pedicle screws were implanted. The vertebral levels treated include L3, L4, L5 and S1. A total of 22 screws were implanted at the L3 level, 66 screws in L4, 68 screws in L5 and 24 screws at the S1 level.

### 3.2 Accuracy

Of the total, 148 screws were classified as grade A (82.10%), 29 screws were classified as grade B (16.20%) and 3 screws were classified as grade C (1.70%). No screws were classified as grade D and E. A total of 177 screws (98.30%) were defined as "clinically acceptable" (A + B) (Table 1). The screw accuracy distribution for the treated vertebral level is described in Table 1.

**Table 1:** Screw accuracy based on vertebral level.

		GRS			Total
		A	B	C	
Vertebra	L3	17	5	0	22
	L4	42	21	3	66
	L5	65	3	0	68
	S1	24	0	0	24
Total		148	29	3	180

ANOVA demonstrated statistically significant results between vertebral level and accuracy (*p* < 0.001).

In particular, post hoc analysis showed a lower accuracy of L4 compared to L5 (*p* < 0.001) and L4 compared to S1 (*p* < 0.001).

### 3.3 Superior facet joint violation

A total of 80 screws were evaluated according to the Yson classification: 78 (97.50%) were classified as grade 0 and 2 (2.50%) were classified as grade 1 due to suspicion of contact with the facet. The grade 1 screws were inserted at the L3 level and the L4 level.

### 3.4 Deviation from pre-operative planning

Of the 180 screws implanted in enrolled patients, 178 were analyzed to study the deviation between the final screw position and pre-operative planning. In particular, 2 screws were not subjected to this evaluation due to the failure to save the planning in the central computer of the navigation system. The deviations from pre-operative planning are described in Table 2.

**Table 2:** Deviation from pre-operative planning based on vertebral level.

Variables	L3	L4	L5	S1	Total
Tip Offset Antero-Posterior (mm)	2,05 ± 2,81	3,19 ± 3,17	3,09 ± 2,68	3,58 ± 2,78	3,07 ± 2,9
Tip Offset Medio-Lateral (mm)	2,3 ± 2	2,26 ± 2,65	2,57 ± 2,19	3,6 ± 2,69	2,59 ± 2,45
Tip Offset Cranio-Caudal (mm)	1,4 ± 1,04	1,53 ± 1,78	1,7 ± 1,74	1,73 ± 1,95	1,6 ± 1,7
Tip Offset 3D (mm)	4 ± 2,89	4,86 ± 3,75	5 ± 3	6,09 ± 3,18	4,99 ± 3,32
Entry Point Offset Antero-Posterior (mm)	3 ± 3,25	3,87 ± 3,6	3,76 ± 2,93	2,96 ± 2,31	3,6 ± 3,1
Entry Point Offset Medio-Lateral (mm)	1,25 ± 1,62	1,77 ± 1,46	1,96 ± 2	2,15 ± 2,05	1,83 ± 1,79
Entry Point Offset Cranio-Caudal (mm)	1,35 ± 0,93	1,45 ± 1,67	1,5 ± 1,69	2,83 ± 1,63	1,68 ± 1,66
Entry Point Offset 3D (mm)	4,18 ± 2,96	5,12 ± 2,72	4,99 ± 3,28	5,12 ± 2,72	4,98 ± 3,22
Angul Axial Offset (°)					3.39 ± 3.63
Angul Sagittal Offset (°)					3.77 ± 3.33

#### 3.4.1 Tip deviation

Regarding the linear parameter, the average deviation of the screw tip on the axial plane in the lateral-medial direction is 2.59 mm ± 2.45 and in the antero-posterior direction is 3.07 mm ± 2.9; on the sagittal plane in the cranio-caudal direction the deviation is 1.6 mm ± 1.7. From these data, the three-dimensional deviation of the screw tip was calculated which corresponds to 4.99 mm ± 3.32. No statistical significance based on vertebral level was demonstrated for tip deviation in any plane (all p > 0.05).

#### 3.4.2 Head deviation

The average deviation of the screw head positioned at the entry point of the pedicle in the axial plane in the lateral-medial direction is 1.83 mm ± 1.79 and in the antero-posterior direction is 3.6 mm ± 3.1. On the sagittal plane in the cranio-caudal direction, the deviation is 1.72 mm ± 1.67. The distance in the three-dimensional space of the screw head at the pedicle entry point is 4.98 mm ± 3.22. A statistical significance was observed only for cranio-caudal deviation based on vertebral level (*p* = 0.002).

#### 3.4.3 Angular deviation

Regarding the angular component, on the axial plane, the deviation is 3.39° ± 3.63°, and on the sagittal plane, it is 3.77° ± 3.33.

#### 3.4.4 GRS and tip deviations

ANOVA reported statistically significant results between GRS and tip deviations in lateral-medial direction, antero-posterior direction and 3D, respectively *p* = 0.005, *p* = 0.01, and *p* = 0.003.

**Fig. 2:**
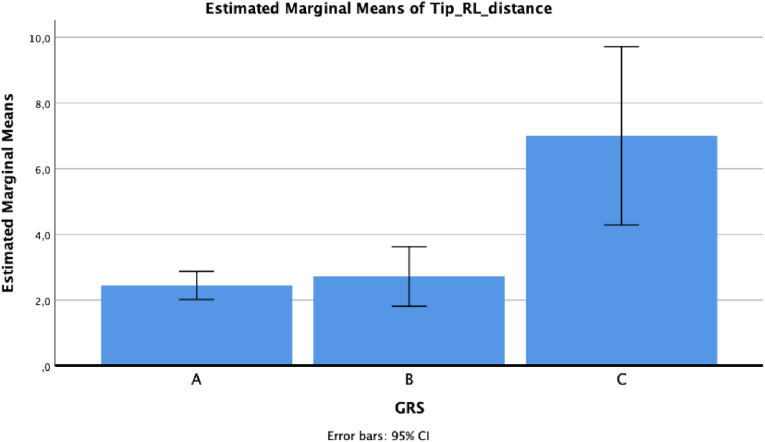
Lateral-medial tip deviation based on GRS grade.

For lateral-medial deviation, the mean value was 2.45 ± 2.36 for grade A, 2.72 ± 2.52 for grade B and 7 ± 1 for grade C (*p* = 0.004 for grade A versus grade C, and *p* = 0.011 for grade B versus grade C, at post hoc analysis) (Fig. 2).

**Fig. 3:**
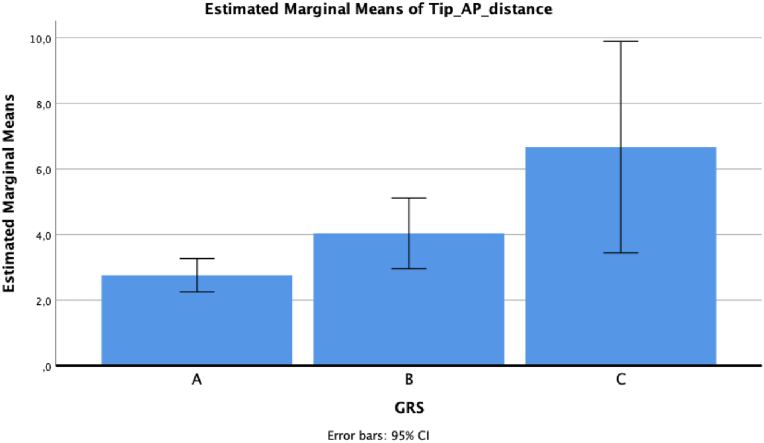
Antero-posterior tip deviation based on GRS grade.

Regarding antero-posterior deviation, the mean value was 2.76 ± 2.55 for grade A, 4.04 ± 3.83 for grade B and 6.67 ± 3.21 for grade C (*p* = 0.05 for grade A versus grade C, at post hoc analysis) (Fig. 3).

In cranio-caudal direction the mean value was 1.6 ± 1.64 for grade A, 1.48 ± 1.89 for grade B, and 3 ± 2.65 for grade C (*p* = 0.344).

**Fig. 4:**
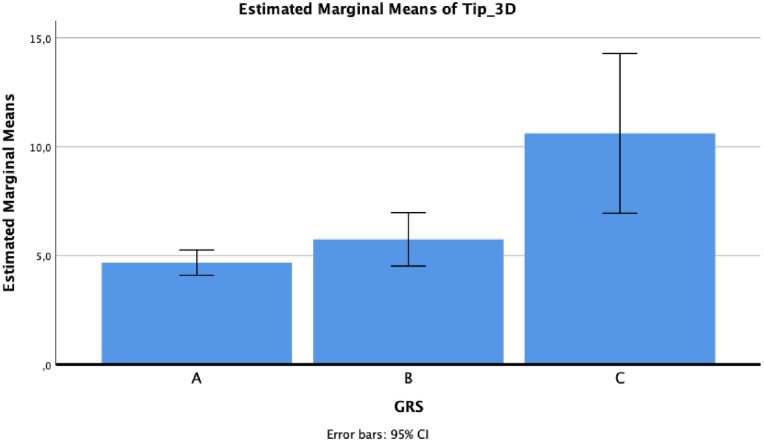
3D tip deviation based on GRS grade.

For 3D deviation, the mean value was 4.68 ± 3 for grade A, 5.75 ± 4.14 for grade B and 10.61 ± 1.77 for grade C (*p* = 0.006 for grade A versus grade C, and *p* = 0.042 for grade B versus grade C, at post hoc analysis) (Fig. 4).

#### 3.4.5 GRS and head deviations

ANOVA reported statistically significant results between GRS and head deviations in antero-posterior direction and 3D, both *p* < 0.001.

For lateral-medial deviation, the mean value was 1.75 ± 1.74 for grade A, 2.26 ± 2.05 for grade B and 1.33 ± 1.15 for grade C (*p* = 0.364).

**Fig. 5:**
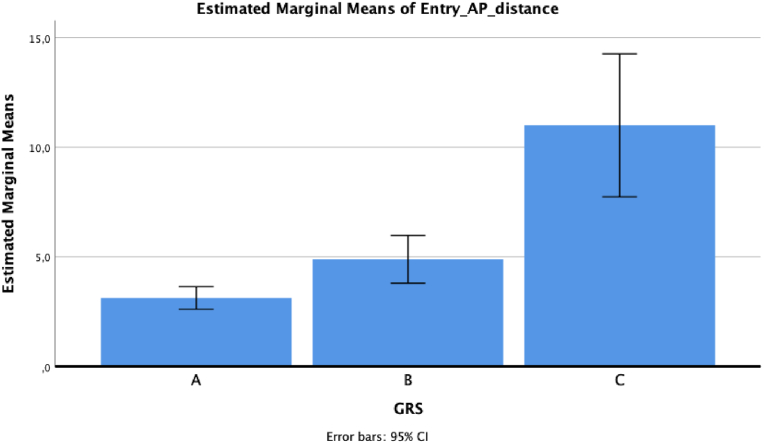
Antero-posterior head deviation based on GRS grade.

Regarding antero-posterior deviation, the mean value was 3.13 ± 2.64 for grade A, 4.89 ± 3.64 for grade B and 11 ± 3.6 for grade C (*p* = 0.013 for grade A versus grade B, *p* < 0.001 for grade A versus grade C, and *p* = 0.002 for grade B versus grade C, at post hoc analysis) (Fig. 5).

In cranio-caudal direction the mean value was 1.7 ± 1.59 for grade A, 1.67 ± 2 for grade B, and 0.67 ± 1.15 for grade C (*p* = 0.568).

**Fig. 6:**
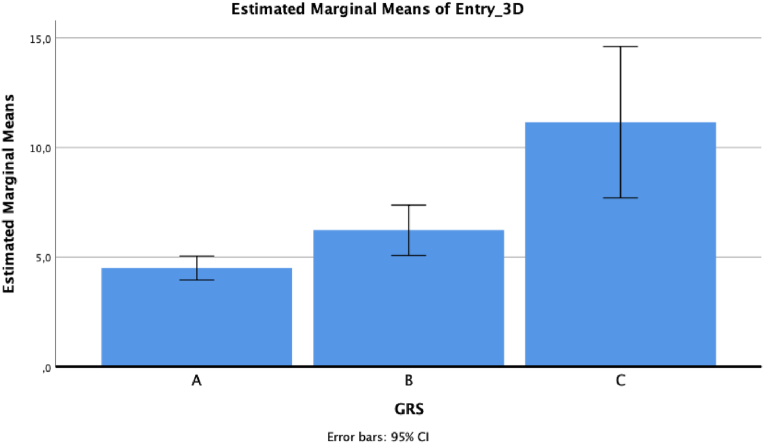
3D head deviation based on GRS grade.

For 3D deviation, the mean value was 4.5 ± 2.83 for grade A, 6.23 ± 3.77 for grade B and 11.15 ± 3.75 for grade C (*p* = 0.025 for grade A versus grade B, *p* = 0.001 for grade A versus grade C, and *p* = 0.025 for grade B versus grade C, at post hoc analysis) (Fig. 6).

## 4 Discussion

The navigation systems were designed and applied in spinal surgery to reduce the mispositioning of the instrumentation and the complications related to screw placement. Several studies demonstrated that navigated and robotic techniques are superior in accuracy and safety compared to conventional techniques (fluoroscopic and freehand techniques) (Papalia et al., 2024; Sun et al., 2020; Feng et al., 2020). The first objective of our study was to evaluate the accuracy and safety in the positioning of pedicle screws using a navigation system guided by intraoperative images acquired with the use of a CBCT. The analysis of the intraoperative control CT showed as 98.30% of the positioned screws were "clinically acceptable" (A + B), and 97.50% of the screws did not breach the facet joint. In this cohort of patients, the use of intraoperative CBCT navigation resulted in a high rate of clinically acceptable screw placement. These results are consistent with previous studies and meta-analyses reporting clinically acceptable screw placement rates of approximately 95–99% with navigated techniques (Papalia et al., 2024). The incremental value of this study is to provide a detailed quantitative analysis of the deviation between planned and implanted screws and its relationship with clinical accuracy. Moreover, the ANOVA test showed a statistically significant correlation between accuracy and vertebral level, demonstrating that the screws achieved a higher accuracy at lower vertebral levels. This correlation can be explained by the position of the reference frame. Indeed, accuracy decreases with increasing distance from the reference frame. In this set of patients, it was placed on the PSIS and upper vertebrae were affected by lower accuracy in screw placement.

The second objective of this study was to evaluate the deviation of the final position of the screw from the intra-operative manually planned. The aim was to evaluate the eventual influence of a possible deviation in the final screw accuracy, and to outline the role of pre-operative planning in spinal surgery. From a clinical perspective, the relevance of screw deviation should be interpreted in relation to established accuracy thresholds. According to the GRS classification, cortical breaches ≤2 mm are considered clinically acceptable. In the present study, despite a mean 3D deviation of approximately 5 mm for both the screw tip and entry point, 98.3% of screws were classified as grade A or B. This finding suggests that deviations of this magnitude did not translate into clinically relevant misplacement. Therefore, within the observed range, deviation from the planned trajectory appears to reflect intraoperative adaptation rather than loss of surgical accuracy. These findings suggest that minor deviations from the planned trajectory should be expected during surgery and do not necessarily indicate technical inaccuracy when overall screw placement remains within clinically acceptable limits. Other studies have already reported high accuracy despite slight deviations from pre-operative planning (Jiang et al., 2020; Gubian et al., 2022; Ha et al., 2023; Godzik et al., 2019; Han et al., 2019; Toossi et al., 2022; Russo et al., 2025).

Our study adopted a linear deviation evaluation system similar to Jiang et al. (2020). They analyzed 254 screws positioned at thoracic and lumbosacral spine with the aid of a robotic arm. They achieved an accuracy of 72% grade A and 28% grade B. The deviation rate was 3.6 mm ± 2.3 for the screw tip and 4.2 mm ± 2.5 for the head. Similar to us, they demonstrated a statistically significant correlation between the deviation rate and the vertebral level, showing that thoracic screws had a lower deviation rate than those positioned at the lumbosacral level. Furthermore, the linear deviation of the screws from the planning did not compromise the clinical accuracy.

Gubian et al. (2022) studied a cohort of 27 patients with a total of 140 screws implanted between L1-S1 using a navigation system. They calculated a 3D linear deviation of the screw head of 5.2 ± 2.4 mm (range 0.5-12.9 mm) and 5.5 ± 2.7 mm (range 1.2-18.3 mm) for the screw tip. They also found a difference between screw deviation and vertebral level, showing a statistically significant correlation between screw direction and screw tip with vertebral level (p < 0.001). Contrary to our results, however, they found no correlation between screw deviation and different degrees of GRS accuracy.

From the results reported in our study and present in the scientific literature, the use of a robotic arm seems to influence screw deviation from the pre-operative planning compared to a navigation system.

Ha et al. (2023) conducted a multicenter retrospective study using a three-dimensional quantitative assessment to investigate the accuracy and safety of a robotic surgical system, the CUVIS-spine (CUREXO Inc., Seoul, Korea). This robotic system produced remarkably low deviation rates, showing an entry offset of 2.86 ± 1.64 mm, a target offset of 2.48 ± 1.74 mm, a depth offset of 1.99 ± 2.13 mm, and an angular offset of 3.07 ± 2.31°. Their study also demonstrated a statistically significant difference in deviation rates between screws implanted with the PLIF approach compared to those implanted with the OLIF approach. Furthermore, they proved that deviations gradually decreased as the operator interventions increased. However, they did not evaluate the 2D linear deviation along the three axes and the correlations between the deviation and the GRS accuracy levels.

A strength of our study was the analysis of screw deviations not only in three-dimensions but also along the three two-dimensional axes and the investigation of any correlations between each deviation and the screw accuracy. From the results, we found a statistically significant correlation between GRS and tip deviations in lateral-medial, anteroposterior, and 3D (p = 0.005, p = 0.01, and p = 0.003, respectively) and between GRS and head deviations in anteroposterior and 3D (both p < 0.001). However, these findings should be interpreted with caution, as only three screws were classified as grade C, resulting in an unbalanced distribution among GRS categories. Therefore, in our cohort the observed deviations were not associated with a clinically relevant reduction in accuracy. However, these statistical results demonstrated that an excessive deviation from the planning can influence the correct positioning of the screws. Furthermore, this quantitative evaluation system can provide very accurate feedback regarding the outcome of the intervention.

There are some limitations to consider in this study. Firstly, although the calculations were performed with cutting-edge software, they were performed manually; while some other studies have used software that automatically calculates the deviation from preoperative planning. Moreover, we included only degenerative cases treated with standard TLIF procedures, in which preoperative planning may primarily provide guidance for trajectory optimization and procedural reproducibility; future studies could evaluate the potential clinical impact of preoperative planning in more complex conditions such as spinal deformity, trauma, or tumor surgery, where anatomical variability and technical difficulty are greater. Furthermore, the interventions were performed by a single experienced spine surgeon using a single surgical approach. All these aspects may limit the generalizability of the results. It would be interesting to perform a comparative study using another surgical technique to further assess the association between deviation rate and accuracy.

## 5 Conclusion

The intraoperatively acquired image navigation system showed high accuracy in pedicle screw insertion. The study of the deviation of the positioned screw compared to the preoperative planning proved the guiding function of the planning during the screw insertion and demonstrated that for slight deviations no decrease in accuracy was found. Finally, the quantitative evaluation method, associated with accuracy classification systems such as the GRS scale, allows for obtaining beneficial and specific information on the outcomes of spinal fusion surgery.

## Ethics approval

The Ethical Committee of the Institution approved the study, which was performed in line with the principles of the Declaration of Helsinki.

## Availability of data and materials

The datasets used and/or analyzed during the current study are not publicly available due to our policy statement of sharing clinical data only on request but are available from the corresponding author on reasonable request.

## Authors’ contributions

Conceptualization, G.V., F.R.; methodology, F.R., G.F.P.; validation, N.N., G.M.; formal analysis, G.F.P., N.N.; data curation, G.F.P., N.N., L.A.; writing—original draft preparation, G.F.P., N.N., G.M.; writing—review and editing G.V., F.R., L.A.; visualization, G.V., R.P.; supervision, R.P., V.D.; funding acquisition, G.V., V.D. All authors have read and agreed to the published version of the manuscript.

## Funding

This research was funded by the Research Grants (BRIC-2022 ID30) of the Italian Workers’ Compensation Authority (INAIL) and PNRR-MAD-2022-12376692, and PNRR-MCNT2-2023-12378359.

## Declaration of competing interest

The authors declare that they have no known competing financial interests or personal relationships that could have appeared to influence the work reported in this paper.
